# Mechanisms of Sodium-Acetate-Induced DHA Accumulation in a DHA-Producing Microalga, *Crypthecodinium* sp. SUN

**DOI:** 10.3390/md20080508

**Published:** 2022-08-09

**Authors:** Yiming Li, Weina Tian, Zhongxiang Fu, Wenqi Ye, Xinwei Zhang, Zhao Zhang, Dongzhe Sun

**Affiliations:** 1 School of Life Sciences, Hebei University, Baoding 071000, China; 2 Key Laboratory of Microbial Diversity Research and Application of Hebei Province, Baoding 071002, China; 3 College of Bioengineering, Beijing Polytechnic, Beijing 100176, China; 4 Institute of Life Sciences and Green Development, Hebei University, Baoding 071000, China

**Keywords:** *Crypthecodinium*, starch, total fatty acid, DHA, acetyl-CoA, organic carbon distribution

## Abstract

Docosahexaenoic acid (DHA) is an omega-3 polyunsaturated fatty acid (PUFA) that is critical for the intelligence and visual development of infants. *Crypthecodinium* is the first microalga approved by the Food and Drug Administration for DHA production, but its relatively high intracellular starch content restricts fatty acid accumulation. In this study, different carbon sources, including glucose (G), sodium acetate (S) and mixed carbon (M), were used to investigate the regulatory mechanisms of intracellular organic carbon distribution in *Crypthecodinium* sp. SUN. Results show that glucose favored cell growth and starch accumulation. Sodium acetate limited glucose utilization and starch accumulation but caused a significant increase in total fatty acid (TFA) accumulation and the DHA percentage. Thus, the DHA content in the S group was highest among three groups and reached a maximum (10.65% of DW) at 96 h that was 2.92-fold and 2.24-fold of that in the G and M groups, respectively. Comparative transcriptome analysis showed that rather than the expression of key genes in fatty acids biosynthesis, increased intracellular acetyl-CoA content appeared to be the key regulatory factor for TFA accumulation. Additionally, metabolome analysis showed that the accumulated DHA-rich metabolites of lipid biosynthesis might be the reason for the higher TFA content and DHA percentage of the S group. The present study provides valuable insights to guide further research in DHA production.

## 1. Introduction

Docosahexaenoic acid (DHA, C22:6), a kind of polyunsaturated fatty acid (PUFA), is an important component of the brain and retina, and thus critical to intellectual and visual development in infants [1]. Deep sea fish oil used to be the major source of DHA production [2], but it has a typical fishy smell and is susceptible to contamination [3]. Besides, deep sea fish oil contains non-negligible amounts of eicosapentaenoic acid (EPA), which is associated with neonatal growth retardation [4]. Nowadays, when combined with mature microalgal fermentation techniques, microalgae have become the primary producers of DHA, especially for *Crypthecodinium*, a strict heterotrophic dinoflagellate in which DHA accounts for approximately 20% to 60% of the total fatty acid (TFA) content and a minimal accumulation of DHA intermediates [1]. Moreover, *Crypthecodinium cohnii* is the first microalgae certified by the Food and Drug Administration (FDA) for DHA production [5].

However, when compared with other oleaginous microalgae, the TFA content is relatively low and only accounts for about 20% of the dry weight in *Crypthecodinium cohnii* [6]. Besides, the intracellular starch content (more than 30% of the dry weight) is usually higher than the lipid content and may compete with the organic carbon involved in lipid accumulation in *Crypthecodinium* sp. SUN [7]. As lipid and starch are two main storage components in microalgae under abiotic stress conditions, their mutual transformation relationship has often been discussed [8]. Studies have shown that the reduction of starch biosynthesis can cause an increase in lipid accumulation in microalgae. When compared with the wild type (WT), a starch-deficient mutant of *Crypthecodinium cohnii* had a decrease in starch content but an increase in lipid and DHA contents by 1.12-fold and 1.13-fold, respectively, of that found in WT [9]. Besides, starch-to-lipid conversion is generally observed in microalgae under stress conditions [10,11,12,13]. High salinity was shown to promote carbon redistribution and starch conversion to lipid accumulation in *Chlorella sorokiniana* [10]. Further studies showed that rapid turnover of starch was likely to provide carbon for fatty acid biosynthesis in *Chlamydomonas debaryana* [13]. Therefore, elucidating the relationship between lipids and starch in *Crypthecodinium* would provide the basis for improving DHA production.

Different carbon sources could alter the lipid and starch composition in microalgae [14]. Glucose and sodium acetate, two common carbon sources, have been proven to lead to the increase of carbohydrate and lipid contents in microalgae, respectively [15,16]. Besides, studies have further investigated the regulatory mechanisms of carbon sources on lipid accumulation in microalgae. Intracellular glucose is the substrate of starch biosynthesis, and the acetyl-CoA formed by acetate is the precursor of fatty acid biosynthesis in microalgae [17,18]. The overexpression of acetyl-CoA synthetase (ACS) could result in an increase in carbon flux toward acetyl-CoA biosynthesis and result in the enhancement of neutral lipid biosynthesis in *Chlamydomonas reinhardtii*, a finding that indicates the importance of precursor supply for lipid accumulation [19]. Thus, it is reasonable to assume that sodium acetate can increase fatty acid accumulation in the heterotrophic *Crypthecodinium* sp. SUN.

Besides, the proportions of fatty acids in microalgae would be affected by factors like temperature, salinity and nutrients. For instance, low temperatures (10–15 °C) increased the proportion of unsaturated fatty acids (PUFAs) in heterotrophic cultures of microalgae, which related to the membrane fluidity [20]. The upregulation of the acetyl-CoA carboxylase (ACCase), type-II fatty acid synthase (FAS) and fatty acid desaturase (FAD) stimulated membrane lipid desaturation [20,21]. Study has shown that compared with lactose, glucose increased the oleic acid (C18:1) proportion (35.7% of TFA) by upregulating the expression of stearoyl ACP desaturase (SAD) in dark-cultured *Chlorella zofingienesis* [22]. Therefore, the intracellular metabolic pathways of different carbon sources may be completely different, resulting in significant accumulation of different types of lipids. However, little is known about the influence of carbon sources on the DHA proportion in *Crypthecodinium* sp. SUN. Thus, the effect of different carbon sources on fatty acid proportions has been investigated in *Crypthecodinium* sp. SUN in the present study. Exploring the causes of their metabolic differences may provide clues for improving TFA accumulation and fatty acid proportion.

*Crypthecodinium* sp. SUN, a newly isolated strain of *Crypthecodinium* that can produce DHA, was used in the present study. Three groups of carbon source (glucose, sodium acetate and mixed carbon sources (G, S and M, respectively)) were used to study the routes of starch and fatty acid accumulation in *Crypthecodinium* sp. SUN. The cell number, dry weight (DW) and glucose consumption were first measured at different time points. Furthermore, the intracellular contents of TFA, DHA, acetyl-CoA, starch and protein were measured to study the effects of carbon sources on starch and TFA accumulation in *Crypthecodinium* sp. SUN. Finally, comparative transcriptome and metabolome analyses were performed to investigate the regulatory mechanisms of intracellular organic carbon distribution in *Crypthecodinium* sp. SUN, which would provide insights into the mechanisms of lipid biosynthesis in this strain.

## 2. Results and Discussion

### 2.1. Effects of Different Carbon Sources on Cell Growth and Glucose Consumption

**Figure 1:**
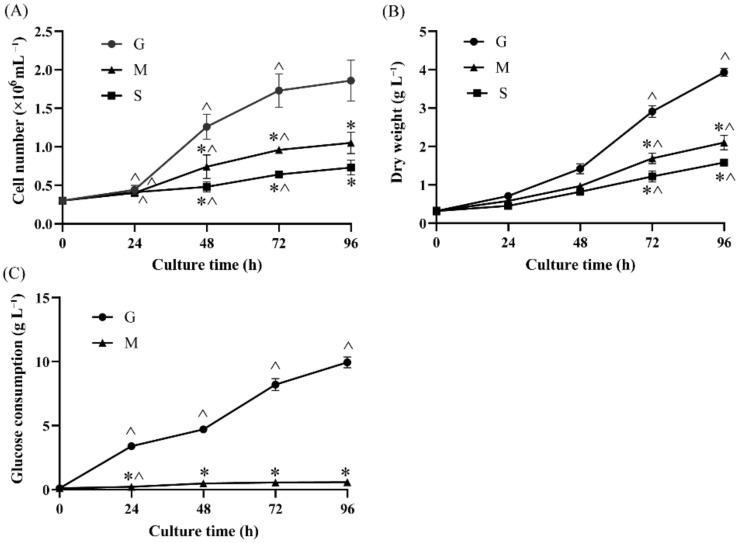
The effect of different carbon sources (G: glucose; M: mixed carbon sources; S: sodium acetate) on cell number (**A**), dry weight (**B**) and glucose consumption (**C**) in *Crypthecodinium* sp. SUN. Each sample was conducted with three biological replicates and the data points are represented as values ± standard deviation (SD). The statistical significance of the results was tested by a two-way repeated measures ANOVA; * significantly different from the G group within each time point (*p* < 0.05); ^ significantly different from the value of the previous time point in the same group (*p* < 0.05).

Different groups of carbon source, including G, S and M were used to study the routes of starch and fatty acid accumulation in *Crypthecodinium* sp. SUN. The cell number and dry weight (DW) were first measured and are shown in Figure 1. The cell number of the three groups all increased through the culturing period and showed obvious differences after 24 h cultivation (Figure 1A). The cell number of G group reached 1.86 × 10^6^ cell mL^−1^ at 96 h, which was 1.77-fold and 2.55-fold of that in the M and S groups, respectively. These results suggest that when compared with sodium acetate, glucose might be the better carbon source for heterotrophic microalgae cultivation and could provide more energy for microalgae growth [23].

The influence of different carbon sources on the DW of *Crypthecodinium* sp. SUN was measured and shown in Figure 1B. The DW of the G group was observably higher than that of the M and S groups and achieved about 3.93 g L^−1^ at 96 h. Consistent with the cell number results, the S group yielded the lowest DW, which was 1.58 g L^−1^ at 96 h, half of that in the G group. The highest biomass of the G group among three groups might have been due to glucose utilization. However, glucose was also found in the M group, but the cell number and DW were also much lower than those in G group. Therefore, the glucose consumption of the M and G groups was measured and is shown in Figure 1C. The glucose consumption by the G group increased dramatically from 0 to 96 h (about 9.95 g L^−1^ at 96 h). In contrast, glucose consumption (0.58 g L^−1^) by the M group only slightly increased (less than 1 g L^−1^) after 96 h of cultivation. These results suggest that sodium acetate might restrict glucose absorption and utilization in *Crypthecodinium* sp. SUN. This interesting phenomenon was first observed in this study, and the subsequent comparative transcriptome and metabolome analyses might help to uncover its underlying mechanisms.

### 2.2. Effect of Different Carbon Sources on Fatty Acid Biosynthesis and DHA Production

**Figure 2:**
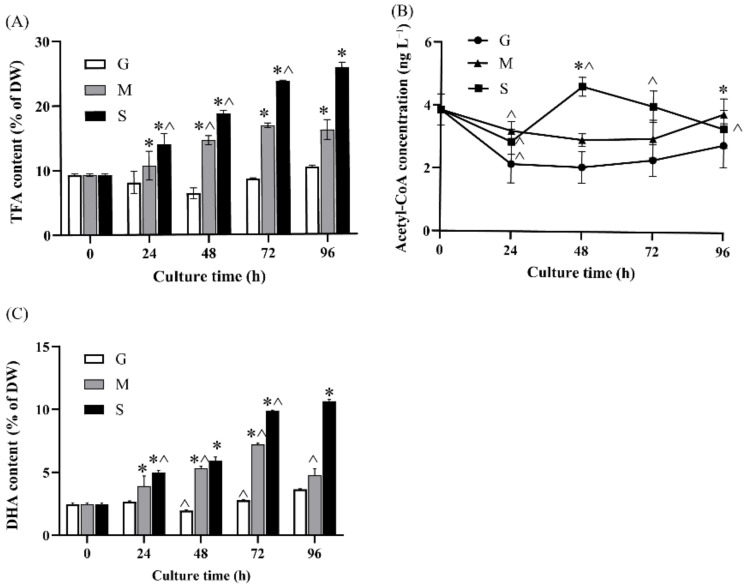
The effect of different carbon sources (G: glucose; M: mixed carbon sources; S: sodium acetate) on total fatty acid (TFA) content (**A**), acetyl-CoA concentration (**B**), and docosahexaenoic acid (DHA) content (**C**) in *Crypthecodinium* sp. SUN. Each sample was tested with three biological replicates, and the data points are represented as values ± SD. The statistical significance of the results was tested by a two-way repeated measures ANOVA; * significantly different from the G group within each time point (*p* < 0.05); ^ significantly different from the value of the previous time point in the same group (*p* < 0.05).

To enhance the lipid accumulation in microalgae, the flow of organic carbon to TFA biosynthesis is usually expected [6,24,25]. Different carbon sources have been shown to alter the lipid and starch composition in microalgae [14]. Therefore, the TFA content and fatty acid profiles in *Crypthecodinium* sp. SUN with the different carbon source groups were measured. As shown in Figure 2A, the TFA content of the S and M groups steadily increased along with culturing time and the TFA concentration first decreased and then increased in the G group. Besides, compared with the G and M groups, the TFA content of the S group was highest among the three groups at each time point. The highest TFA content of the S group was 25.81% of DW at 96 h, which was 1.60-fold and 2.47-fold of that of the M and G groups, respectively. The above results were similar to the results in *Chlorella vulgaris* and *Nannochloropsis oceanica*, in which acetate could increase the fatty acid accumulation [26,27].

Acetyl-CoA formed by acetate is the precursor of fatty acid biosynthesis, and it might be the reason for the high TFA content of the S group. Thus, the cellular acetyl-CoA content of the three groups was measured. As can be seen from Figure 2B, the concentration of acetyl-CoA in the S and M groups had no significant difference with the G group except for the values at 48 and 96 h in the S group. The concentration of acetyl-CoA in the S group was significantly higher than that at 48 and 96 h compared with the G and M groups. Besides, the acetyl-CoA content in the S group was also significantly higher than that in the M group at 48 h. The results indicate that sodium acetate could result in higher cellular acetyl-CoA content. Collectively, the variation trend in acetyl-CoA content among the three groups were coordinated with TFA content, which indicated that sodium acetate could produce more precursors than glucose to support fatty acid biosynthesis, and the cellular acetyl-CoA content might be critical for fatty acid biosynthesis in *Crypthecodinium* sp. SUN.

Different carbon sources were found to not only influence TFA accumulation but also to cause an alteration in fatty acid profiles in *Crypthecodinium* sp. SUN (Table 1). As shown in Table 1, the percentage of saturated fatty acids (SFAs) accounted for about 30% of TFA and had no significant variation from 0 to 96 h. The monounsaturated fatty acids (MUFAs) of the three groups decreased from 41.10% to 34.81%, 34.95% and 27.58% of TFA from 0 to 96 h in the G, M and S groups, respectively. More importantly, the maximum value of the PUFAs of S group (43.41% of TFA) and M group (41.7% of TFA) were much higher than that of the G group (35.50% of TFA). The highest DHA percentage (41.30% of TFA) was found in the S group at 96 h, which was 1.12-fold and 1.18-fold of that in the M and G groups, respectively. These results indicate that in the presence of sodium acetate, the cells would favor PUFA over MUFA accumulation.

As both TFA content and DHA percentage were increased by sodium acetate, the DHA content in the S group was significantly higher than that in the G and M groups (Figure 2C). The DHA content of the S group was gradually increased by time and reached a maximum (10.65% of DW) at 96 h that was 2.92-fold and 2.24-fold of that in the G and M groups, respectively. These results indicate that sodium acetate could significantly increase the DHA accumulation in *Crypthecodinium* sp. SUN.

### 2.3. Effect of Different Carbon Sources on Starch and Protein Contents

**Figure 3:**
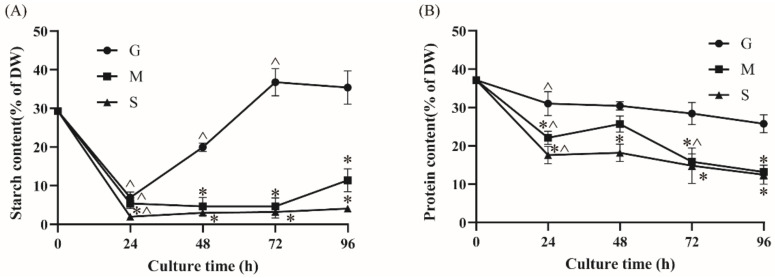
The effect of different carbon sources (G: glucose; M: mixed carbon sources; S: sodium acetate) on the starch content (**A**) and protein content (**B**) in *Crypthecodinium* sp. SUN. Three biological replicates were obtained for each sample. The data points are represented as values ± SD. The statistical significance of the results was tested by a two-way repeated measures ANOVA; * significantly different from the G group within each time point (*p* < 0.05); ^ significantly different from the value of the previous time point in the same group (*p* < 0.05).

In addition to lipids, starch is a major energy storage component in microalgae [14]. Many studies have focused on the metabolic interrelationship between starch and lipid. Previous studies have shown that cells tend to firstly accumulate starch when the carbon source is glucose [25]. In present study, the effect of different carbon sources on starch content was identified. The time course of starch content was measured and is shown in Figure 3A. The G group had the highest starch content in three groups. The starch content of the G group increased dramatically after 24 h cultivation, reaching a maximum (36.79% of DW) at 72 h. As glucose was the direct substrate of starch biosynthesis, it is reasonable that starch was largely accumulated in G group. After 72 h cultivation, the starch content of the glucose group decreased to 35.39% of DW at 96 h. Many studies have shown that the reduction in starch content might be related to lipid accumulation in microalgae [9,10,12]. The decline in starch content after 72 h in group G might be due to the carbon flux flow into other storage components [13]. On the contrary, the starch content of the S group was the lowest among three groups, and the highest starch content was only about 4% of the DW. The starch content in the M group resembled that of the S group but with a slightly higher starch content at each time point. Therefore, it could be concluded that sodium acetate might cause restrictions in starch biosynthesis but could lead to increases in fatty acid accumulation.

Except for starches and lipids, protein is another essential component; it is mainly used as enzymes involved in multiple enzymatic reactions and constitutes structural components in cells [28]. Variations in the protein content in *Crypthecodinium* sp. SUN were measured and are shown in Figure 3B. The highest protein content of the three groups was 37.14% of DW at 0 h after which the protein content in all three groups gradually decreased and reached the lowest level at 96 h. The protein content of the G group at 96 h was 25.77%, which was 1.96-fold and 2.06-fold of that in the M and S groups, respectively. The lower protein content may be due to the higher TFA accumulation in the sodium-acetate-fed groups.

Collectively, contrary to the increase in TFA accumulation, starch and protein contents were significantly restricted in the presence of sodium acetate.

### 2.4. Comparative Transcriptome Analysis under Different Carbon Source Treatments

**Figure 4:**
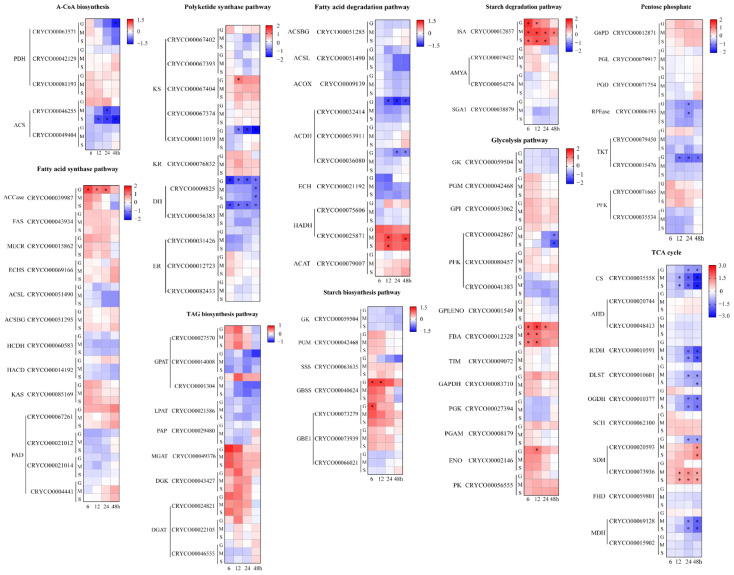
Comparative transcriptome analysis of *Crypthecodinium* sp. SUN cells cultivated with different carbon sources for 6, 12, 24 and 48 h. The heatmap shows the Log_2_ (Fold Change) value of gene expression levels at each time point compared with 0 h. Genes are shown in red (upregulated) and blue (downregulated). See Supplementary file 1: Figure S4 for more details of the RNA-seq data.

To elucidate the regulatory mechanisms of organic carbon distribution in *Crypthecodinium* sp. SUN using different carbon sources, comparative transcriptome analysis was performed at 0, 6, 12, 24 and 48 h. In total, 78,727 expressed genes were detected through RNA-seq analysis, among which 52,414 were known genes and 26,313 were new genes. Up and downregulation of these genes is shown in Supplementary file 2: Table S1. Databases including the Non-Redundant Protein Sequence Database (NR), Swiss-Port Protein Sequence Database (Swiss-Port), Gene Ontology (GO), and Kyoto Encyclopedia of Genes and Genomes (KEGG) were used to annotate all of the transcripts, with the findings supplied in Supplementary file 1: Figure S1. To verify the parallelism of all samples, the principal component analysis (PCA) yielded clear separation among the three groups, which indicated that the parallelism of samples was good (Supplementary file 1: Figure S2). Besides, KEGG enrichment analysis was performed and is shown in Supplementary file 1: Figure S3. Seventeen and three different pathways were enriched by KEGG in G6 versus M6 and G6 versus S6, respectively. Among these pathways, the enrichment of the “Fatty acid biosynthesis”, “Fatty acid degradation”, “TAG biosynthesis”, “Starch biosynthesis” and “Starch degradation” pathways showed that starch and lipid biosynthesis were significantly different based on the carbon source. Besides, glycolysis, tricarboxylic acid (TCA) cycle and pentose phosphate pathways would provide energy, organic carbon intermediate metabolites and reduced nicotinic adenine dinucleotide phosphate (NADPH) for starch, protein and fatty acid biosynthesis. Therefore, specific carbon and metabolic energy pathways, including fatty acid biosynthesis, fatty acid degradation, triacylglycerol (TAG) biosynthesis, starch biosynthesis, starch degradation, glycolysis, TCA cycle and pentose phosphate pathway were specifically analyzed to investigate the regulating mechanisms of intracellular organic carbon distribution. As *Crypthecodinium* possesses a large genome, most of genes in *Crypthecodinium* sp. SUN cells were duplicated. Details of genes with multiple copies are shown in Supplementary file S1, and the copies of key genes in the different pathways with transcripts per million reads (TPM) > 10 were selected and are shown in Figure 4.

#### 2.4.1. Acetyl-CoA Biosynthesis and Lipid Metabolism

As an increase in intracellular acetyl-CoA concentration could induce TFA accumulation in the S group (Figure 2A,B), supplying enough acetyl-CoA might be critical for TFA accumulation in *Crypthecodinium* sp. SUN. The pyruvate dehydrogenase component (PDH) catalyzed acetyl-CoA formation from the pyruvate formed during glycolysis [29,30]. Studies have shown that the upregulation of PDH was a key factor stimulating acetyl-CoA production and lipid accumulation in *Chlamydomonas* sp. JSC4 [12]. However, the comparative transcriptome analysis showed unexpected results, where the expressions of genes coding three PDH isoenzymes had no significant differences among the three groups in the present study (Figure 4). Except for pyruvate, acetyl-CoA can also be generated from acetate catalyzed by acetyl-CoA synthase (ACS), which is a main source of acetyl-CoA in the early lipid accumulation stage [27,31]. Two ACS isoforms (CRYCO00046255 and CRYCO00049404) were found in *Crypthecodinium* sp. SUN, among which, CRYCO00046255 was downregulated in the M and S groups compared with the G group. Thus, the downregulation of PDH and ACS in the S group indicated that rather than changes in expression levels, the supply of the precursor of acetyl-CoA (sodium acetate) is more important for intracellular acetyl-CoA accumulation in *Crypthecodinium* sp. SUN.

Acetyl-CoA carboxylase (ACCase) catalyzes the formation of malonyl-CoA from acetyl-CoA, which is the first step in fatty acid biosynthesis. [32]. Study has shown that overexpression of ACCase could enhance lipid accumulation in *Chlamydomonas reinhardtii* [33]. Unlike the previous study, the expression level of ACCase was downregulated in the S group, but the TFA content was significantly increased compared with the G group (Figure 4). Thus, the expression level of ACCase might not be the key factor affecting TFA accumulation in *Crypthecodinium* sp. SUN.

The fatty acid synthase (FAS) pathway is a key pathway for fatty acid and DHA biosynthesis in microalgae [34,35]. Previous studies have shown that different carbon sources could regulate the expression of key genes in both the FAS pathway in filamentous fungus such as *Glarea lozoyensis*, *Haematococcus pluvialis* and *Nannochloropsis oceanica* [34,36]. However, although sodium acetate significantly increased TFA and DHA accumulation and DHA percentage in *Crypthecodinium* sp. SUN (Figure 2 and Table 1), key genes in the FAS pathway such as fatty acid synthase (FAS) and malonyl enoyl ACP reductase (MECR) showed no significant differences among the three groups (Figure 4). Similarly, genes involved in fatty acid degradation, such as acetyl-CoA synthetase bubblegum (ACSBG), also showed no differences among the three groups (Figure 4). The above results were different from those found in previous studies, in which, along with the stress-induced fatty acid accumulation, genes in the fatty acid biosynthesis pathway were upregulated and in the fatty acid degradation pathway were downregulated [37]. It is worth noting that fatty acid desaturates (FADs) were essential for the polyunsaturated fatty acid (PUFA) biosynthesis [34,38]. Studies have shown that the over-expression of FADs stimulates the PUFA accumulation in *Nannochloropsis oceanica* [39]. However, the expression of fatty acid desaturases (FADs) among the three groups has no significant difference in *Crypthecodinium* sp. SUN (Figure 4). In addition, *Crypthecodinium* sp. SUN belongs to the group, dinoflagellates, which have distinctive nuclear features. Their nucleuses are unusual in terms of their permanently condensed nucleosome-less chromatin, immense genome, low protein-to-DNA ratio, guanine–cytosine-rich methylated DNA and unique mitosis process [40]. Therefore, the regulation pattern of DHA accumulation would be different from that of other oleaginous microalgae, which caused the special phenomenon of increased DHA accumulation but no regular change in the expression of related genes in fatty acid biosynthesis in *Crypthecodinium* sp. SUN. Thus, the expressions of FADs might not be the key factor affecting TFA and DHA accumulation in *Crypthecodinium* sp. SUN.

Except for the FAS pathway, the PKS pathway is another key pathway for DHA biosynthesis in microalgae [41]. Four genes from the PKS pathway were found in *Crypthecodinium* sp. SUN, including 3-ketoacyl synthase (KS), 3-ketoacyl reductase (KR), dehydrase/isomerase (DH/IS) and enoyl reductase (ER). Compared with the G group, the third isoenzyme of the KS gene (CRYCO00067404) was downregulated in the S group, but another isoenzyme, numbered CRYCO00011019, was upregulated in the S group. In addition, the two dehydrase (DH) genes were upregulated in the S and M groups compared with that in the G group, which might be responsible for the increased DHA accumulation in the S group. It was worth noting that the different isozymes of KS, DH and ER genes show different expression patterns at the same time point, which reflected the complexity of the regulatory mechanisms of DHA biosynthesis in the PKS pathway in *Crypthecodinium* sp. SUN.

Triacylglycerol (TAG) has been proved that as the finial storage form of DHA in microalgae [39,40,41]. In this study, six genes participating in TAG biosynthesis were founded through RNA-seq analysis, including glycerol-3-phosphate O-acyltransferase (GPAT), lysophosphatidic acid acyltransferase (LPAT), phosphohydrolase (PAP), 2-acylglycerol O-acyltransferase 2 (MGAT), diacylglycerol kinase (DGK) and diacylglycerol O-acyltransferase (DGAT). GPAT was the rate-limiting enzyme for TAG biosynthesis in microalgae [34]. However, there was no significant difference of the expression of GPAT among the three groups. Similarly, the expression of other genes had no significant differences among the three groups in *Crypthecodinium* sp. SUN, especially for DGAT, which catalyzes triacylglycerol (TAG) formation from diacylglycerol (DAG). These results indicate that, compared with other oleaginous microalgae, the regulatory mechanisms of TAG biosynthesis in *Crypthecodinium* sp. SUN are different.

In summary, most genes in fatty acid biosynthesis, fatty acid degradation and TAG biosynthesis showed no significant differences among the three groups, which indicate that the regulatory mechanisms in *Crypthecodinium* sp. SUN are complex. However, the S group had the highest TFA content and DHA proportion (Figure 2A and Table 1). It is worth noting that the acetyl-CoA concentration of the S group was the highest among the three groups (Figure 2B). Thus, rather than the expression levels of key genes involved in acetyl-CoA biosynthesis and fatty acid biosynthesis, the acetyl-CoA supply is the key limiting factor for fatty acid accumulation in *Crypthecodinium* sp. SUN.

#### 2.4.2. Starch Metabolism

We used glucose and sodium acetate as a carbon source, which were the precursors of starch biosynthesis and fatty acid accumulation in microalgae, respectively [14]. Study has shown that glucose as a carbon source could upregulate the starch biosynthesis pathway in marine microalga *Platymonas helgolandica* [42]. In this study, the sodium acetate limited glucose utilization and the starch accumulation in *Crypthecodinium* sp. SUN (Figure 1C and Figure 3A). Thus, it is reasonable to assume that genes coding starch biosynthesis in the S group would downregulate compared with the G group, affecting the starch accumulation in the S group. As shown in Figure 4, the genes involved in starch biosynthesis containing granule-bound starch synthase (GBSS) and the first 1,4-alpha-glucan branching enzyme (GBE1), which numbered CRYCO00073279, were significantly upregulated in the G group. However, compared with the G group, the expression of these two key genes were downregulated in the S group, indicating that the enhanced starch biosynthesis had occurred in the G group. This result was consistent with the high starch content in the G group as shown in Figure 3A. Starch was the primary energy storage component in microalgae cells, and its degradation is related to lipid accumulation [13]. Studies have shown that the turnover of starch to lipid could increase the lipid accumulation in microalgae [43]. Thus, the genes participating in starch degradation were focused on in *Crypthecodinium* sp. SUN. The results show that there were no significant differences among the three groups, indicating that genes containing ISA, AMYA and SGA1 were not the key factors affecting starch degradation in the three groups.

#### 2.4.3. Glycolysis, Pentose Phosphate Pathway and the TCA Cycle

Energy supply is essential for fatty acid and starch biosynthesis. The results show that sodium acetate limited the glucose utilization (Figure 1C), which will affect the energy metabolism in *Crypthecodinium* sp. SUN. Thus, the glycolysis, pentose phosphate pathway and the TCA cycle, as three critical pathways, were focused on in this study.

Glycolysis, a vital metabolic pathway, can provide energy and intermediate metabolites in cells [32]. The enzymes encoded by glucose kinase (GK), 6-phosphofructokinase (PFK) and pyruvate kinase (PK) are the key rate-limiting enzymes throughout the glycolysis pathway [39]. In the DHA-producing microalga, *Schizochytrium limacinum* SR21, the enhanced activities of GK and GAPDH, related to glycolysis, were benefited for the lipid accumulation in [44]. However, in this study, the expression levels of the GK, PFK and PK genes had no significant difference among three groups, indicating the complexity of genes in the glycolysis pathway.

The pentose phosphate pathway beginning with glucose 6-phosphate can provide NADPH for other pathways, such as fatty acid biosynthesis in microalga cells [44]. Studies have shown that the over-expression of glucose-6-phosphate dehydrogenase (G6PD) and 6-phosphogluconate dehydrogenase (PGD) in the pentose phosphate (PPP) pathway can enhance lipid accumulation by providing NADPH in oleaginous diatom *Fistulifera solaris* [45]. Although *Fistulifera solaris* and *Crypthecodinium* sp. SUN come from two different taxonomic groups, NADPH supply for PPP pathway is essential for fatty acid biosynthesis in both the above microalgae. Sodium acetate increased the TFA accumulation in *Crypthecodinium* sp. SUN (Figure 2A), thus, the expression changes of the PPP pathway were analyzed in the present study. Surprisingly, the key genes, G6PD and PGD, showed no differences among the three groups (Figure 4), which indicated that the expression levels of G6PD and PGD would not be the key limiting factors affecting the NADPH supply for fatty acid accumulation in *Crypthecodinium* sp. SUN. *Crypthecodinium* sp. SUN belongs to the group of dinoflagellates, which have nucleuses that are unusual in terms of their permanently condensed nucleosome-less chromatin, immense genome, low protein to DNA ratio, guanine-cytosine rich methylated DNA and a unique mitosis process [46]. These distinctive nuclear features of *Crypthecondiium* might be the reason for the unique expression patterns of the PPP pathway in *Crypthecodinium* sp. SUN.

The TCA cycle, the main way to obtain energy for cell metabolism, is a common destination of the complete oxidation of starch, lipid and protein products [37]. Studies have shown that carbon sources can affect the energy supply due to the availability of carbon sources [47]. Glucose, as a direct carbon source, can provide more energy in energy metabolism in microalgae compared with sodium acetate [48]. Besides, sodium acetate limited glucose utilization and decreased the biomass in *Crypthecodinium* sp. SUN (Figure 1). Thus, it is reasonable to assume that sodium acetate would downregulate the TCA cycle and provide less energy to support cell growth, compared with glucose. Citrate synthase (CS), a rate-limiting enzyme of TCA cycle, catalyzes the condensation of acetyl-CoA and oxaloacetic acid to form citric acid, which is the first step of TCA cycle [37]. In addition, isocitrate dehydrogenase (ICDH) and 2-oxoglutarate dehydrogenase E1 component (OGDH) are also rate-limiting enzymes in the TCA cycle [37]. As shown in Figure 4, CS, ICDH and OGDH were significantly downregulated in the M and S groups, indicating that downregulation of the TCA cycle in these two groups had occurred. Thus, less energy was produced in the M and S groups, which could have been the reason that the cell number and DW in these two groups were lower than that of the G group (Figure 1A,B).

Finally, 16 genes were selected for real-time quantitative PCR (qPCR) analysis to verify the accuracy of the transcriptome data (Supplementary file 1: Figure S4). Among these genes, ACCase showed significant upregulation in the G group versus the other groups. ACS and OGDH had upregulation in the G group but downregulation in the M and S groups. The expression levels of other genes showed trends resembling the transcriptome data in Figure 4. These results indicated that the transcriptome results were reliable.

In summary, significant downregulation of CS, ICDH and OGDH in the M and S groups in the TCA cycle are related to energy supply and may have been the reason for the lower cell number and DW of the M and S groups versus the G group.

### 2.5. Metabolome Analysis under Different Carbon Source Treatments

The levels of metabolites were affected by the supply of different carbon sources in *Crypthecodinium* sp. SUN. To elucidate the accumulation of different metabolites under different carbon source cultivation, metabolome analysis was performed at 0, 12 and 48 h. A total of 524 metabolites were detected by LC–MS and annotated in the KEGG database (Table S2). PCA was performed to investigate the correlations between metabolite levels and carbon source and showed a clear separation among the three groups, which indicated that the parallelism of samples was good (Figure S5). As shown in Figure S6, 11, 11, 9 and 8 different pathways were enriched by KEGG in G12 versus S12, G12 versus M12, G48 versus M48, and G48 versus S48, respectively. Pathways related to “lipid metabolism”, “TCA cycle” and “Glycerophospholipid metabolism” showed some difference among the three groups. To compare the difference in carbon and energy metabolism among three groups, differential metabolites were defined with VIP > 1 and *p* < 0.05, according to partial least square discrimination analysis (PLS-DA) and orthogonal partial least squares discrimination analysis (OPLS-DA). The key pathways containing “TCA cycle”, “Pentose phosphate” and “Pyruvate metabolism” were specifically analyzed, and a total of 10 identified metabolites were found to be significantly affected by the different carbon sources (Table 2).

Six kinds of metabolites were significantly related to lipid metabolism in *Crypthecodinium* sp. SUN, including phosphatidylcholine (PC 14:0/22:6, 22:6/22:6) and phosphatidylethanolamine (PE 18:1/22:6). These three metabolites all contained DHA and were significantly upregulated in the S group versus the other two groups, indicating that these three metabolites could be induced by sodium acetate. Studies have shown that these metabolites often combine with proteins to form the main components of cell membranes. Besides, these metabolites could also be precursors of diacylglyceride and triacylglyceride (DAG and TAG, respectively) [49]. Thus, the accumulated DHA-enriched intermediates should be the reason for the high TFA and PUFA content in the S group (Figure 2A and Table 1). Through transcriptome analysis, the expression levels of genes in fatty acid biosynthesis were found to be complex and showed no obvious differences among the three groups. However, the metabolites related to lipid accumulation are shown in the S group in Table 2. These results were consistent with the higher TFA accumulation in the S group, and acetyl-CoA as a precursor was found to be critical for TFA accumulation in *Crypthecodinium* sp. SUN.

In the TCA cycle, the most important metabolite was citric acid. The reaction catalyzed by citrate synthase (CS) producing citric acid was a rate-limiting step in the TCA cycle, after which citric acid was converted to aconitic acid. The metabolomic data show that the relative abundance was downregulated in the G group but upregulated in the M and S groups, and the S group had the highest content of citric acid (Table 2). This result indicated that more citric acid accumulation occurred in the S group versus the G and M groups. The transcriptome data show a significant downregulation of the TCA cycle in the S group, a finding that was consistent with citric acid accumulation in the S group (Figure 4 and Table 1). The TCA cycle is an essential pathway that can provide adenosine-triphosphate (ATP) for cell growth in microalgae. The accumulation of citric acid would have induced enough energy in the S group, thus, promoting higher fatty acid accumulation than that found in the G and M groups (Figure 1A,B).

Gluconolactone and gluconic acid (Table 2) are two main metabolites of the pentose phosphate pathway. The relative abundances of gluconolactone and gluconic acid were significantly downregulated in the S group, indicating less accumulation of these two metabolites in the S group. Pentose phosphate begins with glucose 6-phosphate, the S group had no supply of glucose 6-phosphate; thus, the relative abundances of gluconolactone and gluconic acid in the S group were lower than that inf the other two groups. The lower abundance of metabolites along the pentose phosphate pathway might have restricted the supply of NADPH and carbon skeletons for protein biosynthesis and might be the reason for the lower growth rate in the S group.

In summary, the results of the metabolomic analysis showed lipid-related metabolite accumulation in the S group, a finding that was consistent with the higher TFA content in the S group. Besides, the accumulation of critic acid in the S group could contribute more energy for fatty acid metabolism.

## 3. Materials and Methods

### 3.1. Strains and Culture Conditions

The strain used in the present study, *Crypthecodinium*. sp. SUN, has been isolated and screened from the Longhai National Mangrove Natural Reserve (Fujian, China) by our research group previously [25]. Its 18S rDNA sequence can be found on the National Center for Biotechnology Information (NCBI) website (GenBank accession number: KY263646).

*Crypthecodinium* sp. SUN was cultured in modified By+ medium (prepared with freshwater), which consists of 20 g L^−1^ sea salt, 20 g L^−1^ glucose, 1 g L^−1^ tryptone and 1 g L^−1^ yeast extract [25]. The original cell stock was stored in By+ medium at 16 °C away from light. After four days of cell activation, 5 mL of seed culture was inoculated into a new 250 mL Erlenmeyer flask containing 50 mL By+ medium and cultured at 25 °C in a constant temperature shaker incubator with a rotation speed of 150 rpm in the dark [25]. To study the effects of different carbon sources on cell growth, starch content and fatty acid accumulation in *Crypthecodinium* sp. SUN, three groups were set in parallel, namely the G, M and S groups. These three groups had different types and concentrations of carbon source: the G group contained 20 g L^−1^ glucose, the M group contained 10 g L^−1^ glucose and 10 g L^−1^ sodium acetate, and group S contained 20 g L^−1^ sodium acetate. Other nutrients were the same as those in the By+ medium. Samples were taken at 0, 24, 48, 72 and 96 h.

### 3.2. Measurement of Cell Number and Dry Weight

The cell number was counted with a hemocytometer at 0, 24, 48, 72 and 96 h. The samples were diluted with distilled water and shaken as appropriate. Ten microliters of the culture was absorbed and dropped onto the edge of the upper and lower edges of the cover glass located on the hemocytometer and counted under a microscope. All measurements were performed in triplicate.

Five ml of the cultures in the three groups were taken at 0, 24, 48, 72 and 96 h, and centrifuged at 4000× *g* for 5 min to determine the dry weight (DW) [7]. The collected supernatant was used to measure glucose consumption. The precipitated cell pellets were washed twice with distilled water and then filtered with pre-dried and weighed ashless filter papers (Whatman, England, Code No.1440-070). The filter papers were added in a vacuum drying oven (DZF-6050, OLABO, Shanghai, China) and then dried at 80 °C for about 4 h until constant weight [7]. Finally, the DW was calculated by the subtraction method. All measurements were performed in triplicate.

### 3.3. Quantification of Glucose Consumption

The collected supernatant at 0, 24, 48, 72 and 96 h from DW measurement was used to quantify glucose consumption. The 3,5-dinitrosalicylic acid (DNS) method was used to quantify the glucose concentration in the G and M groups, which contained glucose [50]. Then the glucose consumption of the G and M groups was calculated using Equations (1) and (2), respectively. All measurements were performed in triplicate.

Glucose consumption (g L^−1^) = 20 g L^−1^ − Glucose concentration (g L^−1^) (1)

Glucose consumption (g L^−1^) = 10 g L^−1^ − Glucose concentration (g L^−1^) (2)

### 3.4. Fatty Acid Analysis

To methylate fatty acids, 20 mg of lyophilized algal powders were weighed into a covered glass tube. One milliliter toluene and 2 mL 1% sulfuric acid dissolved in methanol (*v*/*v*, 0.05% BHT was added to prevent lipid oxidation) were firstly added to the glass tube. Then, 0.5 mL heptadecanoic acid (C17:0, 1 mg mL^−1^ dissolved in *n*-hexane) was added to the glass tube as an internal standard for fatty acid methyl ester (FAME) analysis. The sealed glass tube was incubated at 85 °C for 2.5 h and oscillated every 30 min in order for the reaction to be complete. After cooling, 1 mL 0.75% sodium chloride solution was added to the glass tube and mixed well. Then, 2 mL *n*-hexane was used to extract FAMEs, the supernatants were collected after centrifugation at 5000× *g* for 5 min and dried with a pressured nitrogen blowing concentrator (LC-DCY-12SY, LICHEN, China), and then the volume was fixed with *n*-hexane to 1 mL. After filtering with a disposable microfilter (13 mm × 0.22 µm, Nylon 66, JINTENG, China), the filtered samples were analyzed on an Agilent 7890A gas chromatograph (GC) equipped with a DB-23 capillary column (30 m × 0.25 mm × 0.25 µm, Agilent, Santa Clara, CA, USA). Nitrogen and synthetic air were used as the carrier gas and make-up gas, respectively. For FAME analysis, the initial injector temperature and column temperature were set at 250 °C and 150 °C, respectively. The column temperature subsequently rose to 200 °C at 10 °C min^−1^ followed by holding at 200 °C for 4 min. All measurements were performed in triplicate.

### 3.5. Extraction and Quantification of Intracellular Acetyl-CoA

The extraction method of acetyl-CoA was modified based on the research of Avidan et al. [51]. About 2 × 10^6^ cells were collected at 0, 24, 48, 72 and 96 h and then quenched with 1 mL pre-cooled acetonitrile: isopropyl alcohol (*v*/*v* = 3:1). The mixture was thoroughly ground in an ice bath for 5 min followed by the addition of 1 mL 0.1 M potassium phosphate buffer (pH 6.7), during which acetyl-CoA was extracted. After centrifugation at 5000× *g* at 4 °C for 5 min, the supernatant was collected for acetyl-CoA determination.

The intracellular acetyl-CoA concentration was quantified with a plant acetyl-CoA enzyme-linked immunoassay (ELISA) kit (Mosak, Code No. Kt 40577). The extracted acetyl-CoA from microalgae cells was coated with sealing membrane and incubated at 37 °C for 30 min after which it was used as the solid phase antibody. Acetyl-CoA was successively added to the microporous membrane coated with monoclonal antibody in advance, and then combined with horseradish peroxidase (HRP)-labeled acetyl-CoA antibody in the kit to form an antibody–antigen–enzyme-conjugate antibody complex. The 96-well ELISA plate was cleaned with the wash solution in this kit, and tetramethyl benzidine (TMB) was added to produce color at 37 °C for 15 min. After addition of acid stop solution, the blue color in the 96-well ELISA plate changed immediately to yellow. The absorbance (optical density (OD)) of each well was measured at 450 nm within 15 min. Finally, the concentration of acetyl-CoA in all samples was calculated according to the standard curve. All measurements were performed in triplicate and each hole was reperforated.

### 3.6. Quantification of Starch

The starch content of all samples was quantified according to the method by Zhang et al. [52]. About 50 mg of lyophilized algae cells were collected at 0, 24, 48, 72 and 96 h, respectively. Then, the lyophilized algae cells in the three groups were accurately weighed and thoroughly ground in a mortar under liquid nitrogen protection. After washing twice in an ice bath with 20 mM Tris/HCl buffer (pH 6.9), a low temperature was maintained during washing. The microalgal residue was collected by centrifugation at 4 °C at 10,000× *g* for 10 min. Five milliliters of an ethanol solution (80%) was added to the algal residue followed by placing in a water bath at 85 °C for 5 min to remove glucose and maltose. Five milliliters of an ethanol solution (80%) was added again to the system, then mixed and cooled on ice. After centrifuging at 12,000× *g* at 4 °C for 10 min, the algal residue was collected and the supernatant was discarded. Then, 2 mL dimethyl sulfoxide (DMSO) was added to the microalgal residue, dissolved by full oscillation and incubated in a boiling water bath for 5 min. Four mL Tris/HCl buffer (20 mM, pH 6.9) and 20 μL thermally stabilized α-amylase were added to the system and boiled again for 5 min. After cooling on ice, 1 mL starch trans-glucosidase solution (1 g L^−1^) and 2 mL sodium acetate solution (pH 4.4) were added to the system. After being shaken well, the samples were incubated in a shaking bath at 60 °C for about 15 min. Then, the samples were centrifuged at 8000× *g* for 5 min and the supernatants were collected to quantify the starch content. The reducing glucose concentration in the supernatant was measured via the 3,5-dinitrosalicylic acid (DNS) method [50]. Starch content was calculated after centrifugation at 4000× *g* at room temperature for 15 min. All measurements were performed in triplicate.

### 3.7. Quantification of Protein

To quantify protein content, 2 mg of lyophilized algal cells was collected at 0, 24, 48, 72 and 96 h. Then, the lyophilized algae cells in the three groups were weighed and ground thoroughly in a mortar. One mL 3% potassium hydroxide solution was added to the samples and then they were incubated at 80 °C for 10 min, followed by centrifugation at 900× *g* for 5 min, and the supernatant was collected into new centrifuge tubes. For more complete collection of all proteins, the sediment was washed three times with distilled water. After centrifugation, all protein in the collected supernatant was detected with the bicinchoninic acid (BCA) Protein Measurement Kit (Beyotime Institute of Biotechnology, Code No. P0012). The mixture of BCA reagents, A and B (*v*/*v*, 30:1), was used to prepare the BCA working fluid (50:1). The samples were incubated at 60 °C for 30 min after adding the BCA working solution. The absorbance of all samples was measured at 562 nm [53]. All measurements were performed in triplicate.

### 3.8. Total RNA Extraction, Quality Control, and cDNA Library Construction

The algae cell cultures were collected in RNA-free centrifuge tubes at 0, 6, 12, 24 and 48 h. The supernatant was discarded after centrifugation at 5000× *g* at 4 °C for 3 min. The precipitated cells were rapidly frozen with liquid nitrogen. The collected algal cells were thoroughly ground under the protection of liquid nitrogen and then transferred to an RNA-free centrifuge tube. The total RNA of *Crypthecodinium* sp. SUN was extracted using RNAiso plus reagent (Takara, Beijing, China, Code No.9108/9109) according to the instructions. To detect the quality of the extracted RNA, a 2100 Bio-Analyzer (Agilent, Santa Clara, CA, USA) and a Nanodrop-2000 (Nano Drop Technologies, Wilmington, DE, USA) were used. To improve the accuracy of the subsequent sequencing, high-quality RNA samples (OD260/280 = 1.8–2.2, OD260/230 2.0, RIN 6.5, 28S:18S 1.0, & gt; 1 μg) were used to construct the sequencing library.

About 1 μg of total RNA was used for the construction of the RNA-seq transcriptome library. The mRNA was isolated using oligo (dT) beads following poly A selection and subsequently segmented with buffer solution. Then, the Super Script double-stranded cDNA synthesis kit (Invitrogen, Waltham, MA, USA, Code No.11917-020) was used for cDNA synthesis. According to Illumina construction principles, cDNA terminal repair, phosphorylation and addition of “A” base were carried out. About 300 bp fragments were selected for PCR amplification and then sequenced after TBS380 quantitation (Illumina HiSeq xten/NovaSeq 6000 sequencer, Illumina, Shanghai, China) to obtain the RNA-SEQ sequencing library.

### 3.9. Read Mapping, Differential Genes Expression Analysis, and Functional Enrichment

The clean reads were aligned to the *Crypthecodinium* sp. SUN reference genome using Top-Hat version 2.0.0 software. The expression level of each transcript was calculated according to the transcripts per million reads (TPM) method. RNA-Seq by expectation maximization was used to quantify gene abundances [54]. In order to distinguish the differentially expressed genes, DESeq2 [55]/EdgeR [56] with Q value ≤ 0.05 and DEGs with |log2FC| > 1 were considered as the screening standard. For further analysis, functional-enrichment analyses, including Gene Ontology (GO) and Kyoto Encyclopedia of Genes and Genomes (KEGG) were performed. GO functional enrichment and KEGG pathway analysis were carried out by Goatools and KOBAS.

### 3.10. Real-Time Quantitative PCR (qPCR)

Total RNA was extracted and reversely transcribed into cDNA (Invitrogen, USA, Code No.11917-020). To verify the transcriptome data, real-time quantitative PCR detecting system (qPCR) was performed using a CFXTM Real-Time System (Bio-Rad, Hercules, CA, USA). Sixteen genes were selected, and the primers were designed using Primer Premier 5 software. The cDNA was amplified by TB Green Premix Ex TaqTMII (Takara, Kyoto, Japan). Three PCR reactions were performed for each sample. Each reaction system contained 10 μL TB Green Premix Ex Taq Ⅱ, 0.8 μL PCR forward and reverse primers (10 mM), 0.4 μL ROX reference dye (50×), 2 μL DNA template, and 6 μL sterile water. The total volume of the formulated system was 20 μL. The PCR cycle was 95 °C for 30 s followed by 40 cycles of 95 °C for 3 s, and 60 °C for 30 s. Relative gene expression was evaluated using the 2^−^^ΔΔCT^ method described by Sirikhachornkit et al. [57].

### 3.11. Metabolite Extraction, LC-MS Analysis, and Metabolite Identification

About 1 × 10^7^ cells collected at each time point were centrifuged at 5000× *g* at 4 °C for 3 min, then the supernatant was discarded. After which the pelleted cells were rapidly frozen and ground with a frozen tissue grinder for 6 min (−10 °C, 50 Hz). About 400 µL of methanol: water = 4:1 (*v*:*v*) extract was added to the ground sample to extract all metabolites, including 0.02 mg mL^−1^ internal standard (L-2-chlorophenylalanine). The mixture was ultrasonically extracted at 4 °C for 30 min and then left standing at −20 °C for 30 min. After centrifugation at 13,000× *g* at 4 °C for 15 min, the supernatant was transferred to sample bottles, and the metabolites were determined and identified by liquid chromatography–mass spectrometry (LC-MS) with an ACQUITY UPLC HSS T3 column (100 mm × 2.1 mm, 1.8 µm; Waters, Milford, MA, USA) as the chromatographic column. Two mobile phases were used in LC-MS analysis, the mobile phase A consisted of 95% water and 5% acetonitrile (containing 0.1% formic acid), and mobile phase B contained 47.5% acetonitrile + 47.5% isopropanol +5% water (containing 0.1% formic acid). Each sample had a 2 μL injection volume, and the column temperature was 40 °C. All extracted metabolites were ionized by electrospray ionization, and the positive (+) and negative (−) ion scanning modes were applied to collect the mass spectrum signals. The scan type (*m*/*z*) of mass spectrometry was 70-1050. Heater and capillary temperatures were 425 and 325 °C, respectively. The positive and negative spray voltages were +3500 and −3500 V, respectively.

The raw data were imported into the metabolomics processing software Progenesis QI (Waters Corporation, Milford, USA). After a series of data processing steps, including baseline filtering, peak identification, integration, retention time correction and peak alignment, the key data matrix was obtained. Then, the software was used to identify characteristic peaks to search the database, and the mass spectrometry information was matched with the metabolic database to facilitate the analysis of metabolomics.

### 3.12. Statistical Analysis

All the experiments were conducted in three biological replicates, and the results in Figures and Tables are expressed as mean value ± standard deviation (SD). The significance of the results was calculated using two-way repeated measures analysis of variance (ANOVA) with IBM SPSS Statistics 26.0. The results were performed with the Bonferroni correction for the *p* value.

## 4. Conclusions

In this study, the heterotrophic *Crypthecodinium* sp. SUN was cultured with different carbon sources. Glucose favored cell growth and starch accumulation. Sodium acetate limited starch accumulation but significantly increased TFA accumulation and DHA percentage, which resulted in a 2.92-fold higher DHA content in the S group than the G group at 96 h. Rather than the expression levels of key genes in fatty acid biosynthesis, the increased intracellular acetyl-CoA content appeared to have promoted fatty acid accumulation. Besides, the accumulation of DHA-rich intermediates in the lipid biosynthesis pathway might be the reason for the higher TFA content and DHA percentage of the S group. In summary, the present study provides valuable insights to guide further research in DHA production.

## Figures and Tables

**Table 1:** The percentage of fatty acid profiles (% of total fatty acid (TFA)) with different carbon sources in *Crypthecodinium* sp. SUN ^#^.

Time (h)	Group ^^^	C14:0	C16:0	C16:1	C18:0	C18:1	DHA	SFAs ^a^	MUFAs ^b^	PUFAs ^c^
0	G	2.63 ± 0.22	13.94 ± 0.62	34.24 ± 1.19	14.82 ± 1.19	6.86 ± 0.27	26.40 ± 0.03	32.14 ± 0.39	41.10 ± 0.93	26.76 ± 1.21
24	G	2.84 ± 0.02	11.74 ± 0.02	30.22 ± 0.99	13.19 ± 0.73	5.76 ± 0.01	34.56 ± 0.05	28.57 ± 3.34	35.98 ± 4.39	35.45 ± 6.63
	M	3.42 ± 0.07 *	16.83 ± 0.38 *	25.50 ± 0.59 *	8.46 ± 0.23 *	7.78 ± 0.02	36.32 ± 0.81	29.63 ± 0.22	34.48 ± 1.82	36.62 ± 3.75
	S	3.04 ± 0.10 *	18.13 ± 0.17 *	25.62 ± 0.79 *	6.78 ± 0.59 *	8.86 ± 0.01 *	36.04 ± 0.15	28.91 ± 2.18	33.28 ± 0.50	37.09 ± 0.71
48	G	3.22 ± 0.05	13.77 ± 0.01	32.06 ± 0.48	12.57 ± 0.37	5.95 ± 0.00	31.04 ± 0.07	30.15 ± 2.54	38.02 ± 2.70	31.83 ± 5.20
	M	3.91 ± 0.02	19.50 ± 0.04 *	22.28 ± 0.49 *	5.31 ± 0.37 *	7.36 ± 0.02	39.71 ± 0.15 *	30.13 ± 0.99	32.13 ± 1.18 *	35.86 ± 1.99 *
	S	3.56 ± 0.02	18.11 ± 0.12 *	25.24 ± 0.32 *	9.13 ± 0.28 *	6.89 ± 0.02	35.40 ± 0.30	32.01 ± 0.81	29.64 ± 1.72 *	40.22 ± 2.72 *
72	G	3.21 ± 0.01	17.95 ± 0.02	29.34 ± 0.05	8.44 ± 0.02	7.40 ± 0.01	32.18 ± 0.05	30.50 ± 0.09	36.73 ± 0.30	32.77 ± 0.32
	M	2.95 ± 0.01	18.52 ± 0.02	22.11 ± 0.23 *	4.83 ± 0.18 *	6.76 ± 0.01	42.83 ± 0.09 *	27.72 ± 0.51	28.95 ± 2.40 *	39.66 ± 3.53 *
	S	4.00 ± 0.06 *	18.98 ± 0.05	22.49 ± 1.36 *	7.15 ± 1.00	6.46 ± 0.00	39.23 ± 0.04 *	31.38 ± 1.13	28.87 ± 0.75 *	43.41 ± 1.26 *
96	G	3.47 ± 0.01	19.15 ± 0.01	27.06 ± 0.11	6.28 ± 0.08	7.75 ± 0.01	34.99 ± 0.04	29.69 ± 0.42	34.81 ± 0.35	35.50 ± 0.76
	M	1.84 ± 0.08 *	15.87 ± 0.62 *	26.95 ± 1.49	8.52 ± 0.80	8.00 ± 0.02	36.84 ± 1.42	27.69 ± 1.72	27.58 ± 0.77 *	41.70 ± 1.07 *
	S	3.92 ± 0.02 *	19.64 ± 0.09	21.00 ± 0.39 *	5.77 ± 0.27	6.58 ± 0.01	41.30 ± 0.15 *	30.72 ± 0.31	34.95 ± 2.28	37.36 ± 4.00

^#^ Explanation for the fatty acid abbreviation notation: C14:0: myristic acid; C16:0: palmitic acid; C16:1: palmitoleic acid; C18:0: stearic acid; C18:1: oleic acid; DHA: docosahexaenoic acid. ^^^ The G, M and S group stand for glucose, mixed carbon sources and sodium acetate, respectively. ^a^ The sum of the percentage of saturated fatty acids (% of TFA). ^b^ The sum of the percentage of monounsaturated saturated fatty acids (% of TFA). ^c^ The sum of the percentage of polyunsaturated fatty acids (% of TFA). * Significantly different from the G group within each time point (*p* < 0.0167 with Bonferroni correction).

**Table 2:** Overview of the differentially produced intracellular metabolites detected in *Crypthecodinium*. sp. SUN cultivated with different carbon sources.

Pathway	Metabolite	Group	Log_2_FC ^#^		VIP		*p* Value	
			12 h	48 h	12 h	48 h	12 h	48 h
Lipid metabolism	Lyso PC (14:0/0:0)	G	0.77	1.69	0.51	1.51	0.0002	0.0000
		M	2.90	2.26	1.65	1.34	0.0000	0.0000
		S	2.13	1.58	1.20	0.93	0.0000	0.0018
	Lyso PC (16:0)	G	1.15	−0.57	5.09	3.43	0.0000	0.0250
		M	1.00	−0.82	4.62	2.98	0.0001	0.0068
		S	1.22	−1.20	5.46	3.66	0.0000	0.0027
	Lyso PC (22:6)	G	1.26	0.78	9.93	11.10	0.0000	0.0000
		M	1.60	1.74	12.17	13.97	0.0000	0.0000
		S	1.35	1.30	10.80	9.70	0.0000	0.0113
	PC (14:0/22:6)	G	9.87	0.30	1.25	0.02	0.0001	0.5223
		M	0.30	0.56	0.02	0.01	0.5223	0.5691
		S	11.26	5.25	2.11	0.18	0.0001	0.2076
	PE (18:1/22:6)	G	4.83	1.24	2.29	0.78	0.0181	0.0346
		M	4.48	−0.14	2.19	0.05	0.0035	0.8663
		S	5.07	0.69	2.83	0.28	0.0025	0.2742
	PC (22:6/22:6)	G	4.87	0.39	2.05	0.16	0.0000	0.5500
		M	4.95	−2.48	2.14	0.32	0.0000	0.0245
		S	5.25	1.17	2.41	0.33	0.0000	0.1300
Energy metabolism	Citric acid	G	−2.08	−0.50	1.66	1.62	0.0000	0.0000
		M	0.47	0.96	1.18	1.99	0.0000	0.0000
		S	0.64	1.38	1.44	2.72	0.0000	0.0000
Pyruvate metabolism	S- Lactoylglutathione	G	−6.02	0.33	5.47	3.64	0.0000	0.0149
		M	−4.09	−4.57	5.48	5.82	0.0000	0.0000
		S	−4.06	−5.03	5.53	6.15	0.0000	0.0000
Pentose phosphate pathway	Gluconolactone	G	−1.42	−0.80	1.71	2.27	0.0000	0.0000
		M	−1.18	−1.34	1.66	1.77	0.0000	0.0001
		S	−1.78	−2.51	1.92	2.28	0.0000	0.0000
	Gluconic acid	G	0.75	1.29	1.10	2.60	0.0000	0.0000
		M	−1.38	0.35	1.09	0.37	0.0000	0.4299
		S	−2.36	−0.56	1.27	0.70	0.0000	0.0286

^#^ The Log_2_FC stands for the relative abundance at each time point compared with that of the G group at 0 h.

## Data Availability

The data presented in this study are available in the Supplementary Materials.

## Supplementary Materials

The following supporting information can be downloaded at: https://www.mdpi.com/article/10.3390/md20080508/s1, Figure S1: Number and distribution of annotated genes in the NR, Swiss-Port, Pfam, COG, GO and KEGG databases; Figure S2: PCA score plot showing transcriptomic separation of sets of three replicates of *Crypthecodinium* sp. SUN cultures at 0, 12, 24 and 48 h in different carbon sources: glucose (G), mixed (M) and sodium acetate (S); Figure S3: KEGG enrichment analysis of the different genes of different groups in *Crypthecodinium* sp. SUN; Figure S4: The differentially expressed genes detected by RT-qPCR at 6 h; Figure S5: PCA score plot showing metabolomic separation of sets of six replicates of *Crypthecodinium* sp. SUN cultures at 0, 12 and 48 h in different carbon sources: glucose (G), mixed (M) and sodium acetate (S); Figure S6: KEGG enrichment analysis of the different metabolites of different groups in *Crypthecodinium* sp. SUN. (A)~(D) were KEGG analysis of S12vsG12, M12vsG12, M48vsG48 and S48vsG48, respectively; Table S1: Overview of DEG in different carbon sources in *Crypthecodinium* sp. SUN; Table S2: Overview of all numbers of metabolites and different numbers of metabolites among the three groups in *Crypthecodinium* sp. SUN; Supplementary file 3: The RNA-seq data of genes involved in selected biological pathways.
