# Supplementary material for: Mechanisms of Sodium-Acetate-Induced DHA Accumulation in a DHA-Producing Microalga, Crypthecodinium sp. SUN
Source: Mar Drugs. 2022 Aug 9;20(8):508. doi: 10.3390/md20080508 (PMC9409966; doi:10.3390/md20080508)
Supplement: Supplementary file 1 [file marinedrugs-20-00508-s001.zip › Supplementary file 1.pdf]

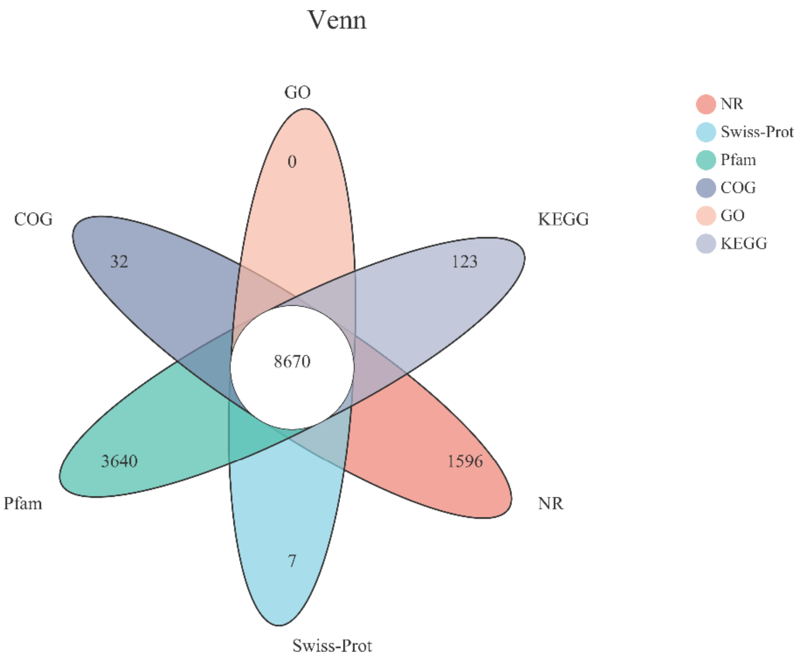

Figure S1: Number and distribution of annotated genes in the NR, Swiss-Port, Pfam, COG, GO and KEGG databases.

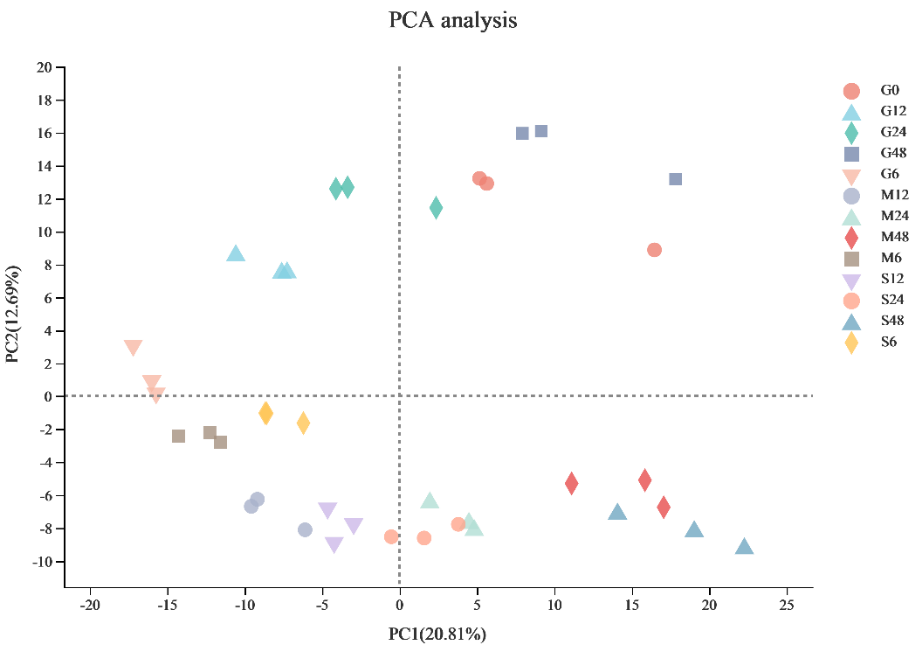

Figure S2: PCA score plot showing transcriptomic separation of sets of three replicates of *Crypthecodinium* sp. SUN cultures at 0, 12, 24 and 48 h in different carbon sources: glucose (G), mixed (M) and sodium acetate (S).

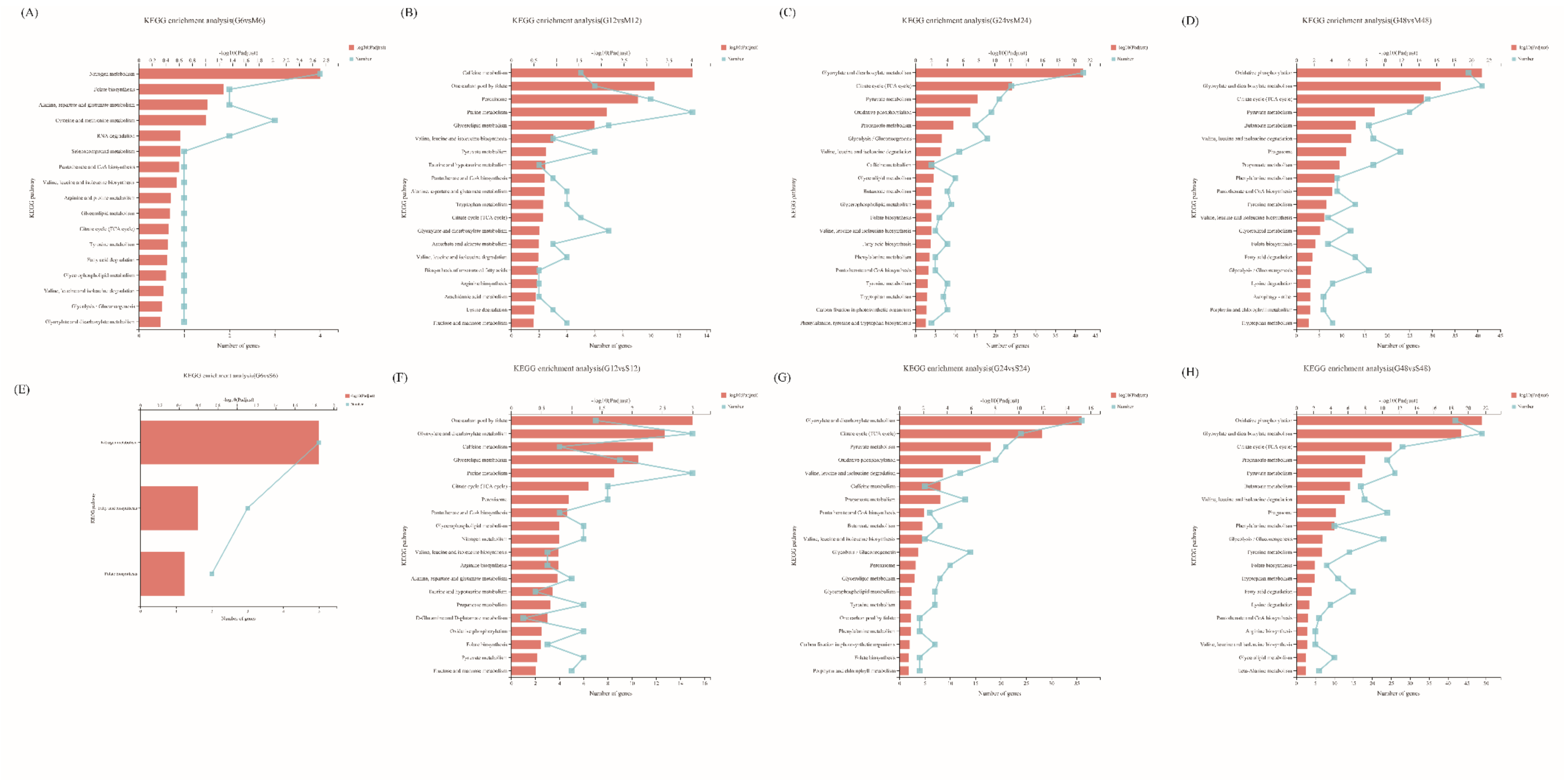

Figure S3: KEGG enrichment analysis of the different genes of different groups in *Crypthecodinium* sp. SUN. (A) ~ (H) were KEGG analysis of G6vsM6, G12vsM12, G24vsM24, G48vsM48, G6vsS6, G12vsS12, G24vsS24 and G48vsS48 respectively.

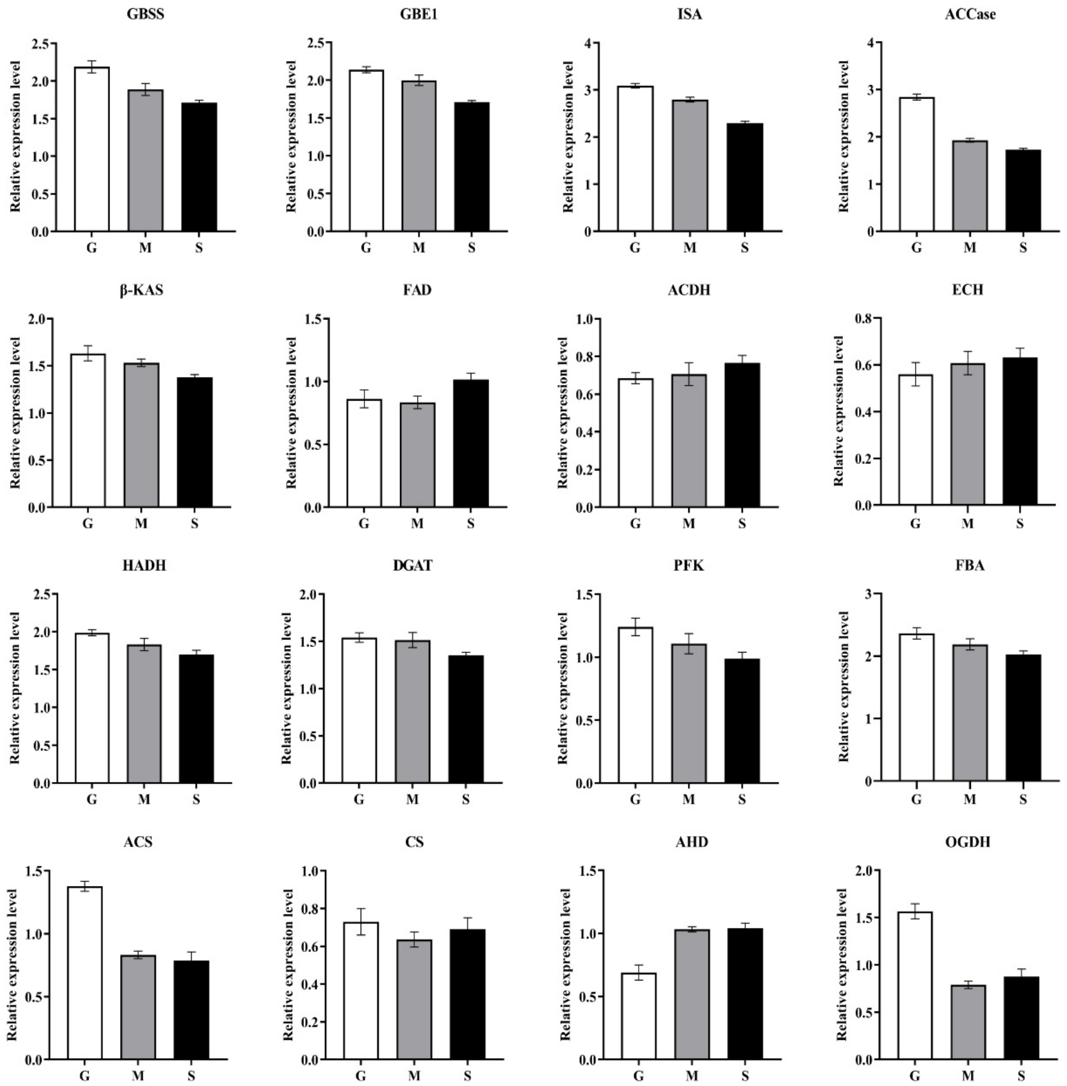

Figure S4: The differentially expressed genes detected by RT-qPCR at 6 h. Experiments were conducted with three biological replicates. The data points were represented as values ± SD. GBSS: granule-bound starch synthase, GBE1: 1,4-alpha-glucan branching enzyme, ISA: iso-amylase, ACCase: acetyl-CoA carboxylase, β-KAS: beta-ketoacyl synthase, FAD: fatty acid desaturase, ACDH: acyl-CoA dehydrogenase, ECH: enoyl-CoA hydratase, HADH: 3-hydroxyacyl-CoA dehydrogenase, DGAT: diacylglycerol O-acyltransferase, PFK: 6-phosphofructokinase, FBA: fructose-bisphosphate aldolase, class I, ACS: acetyl-CoA synthetase, CS: citrate synthase, AHD: aconitate hydratase, OGDH: 2-oxoglutarate dehydrogenase E1 component

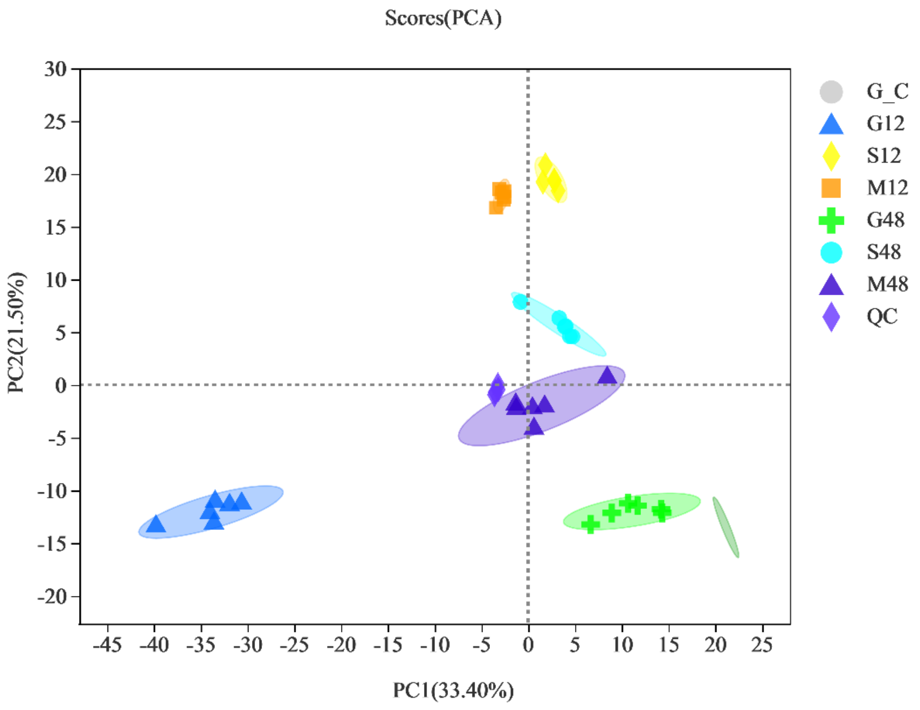

Figure S5: PCA score plot showing metabolomic separation of sets of six replicates of *Crypthecodinium* sp. SUN cultures at 0, 12 and 48 h in different carbon sources: glucose (G), mixed (M) and sodium acetate (S).

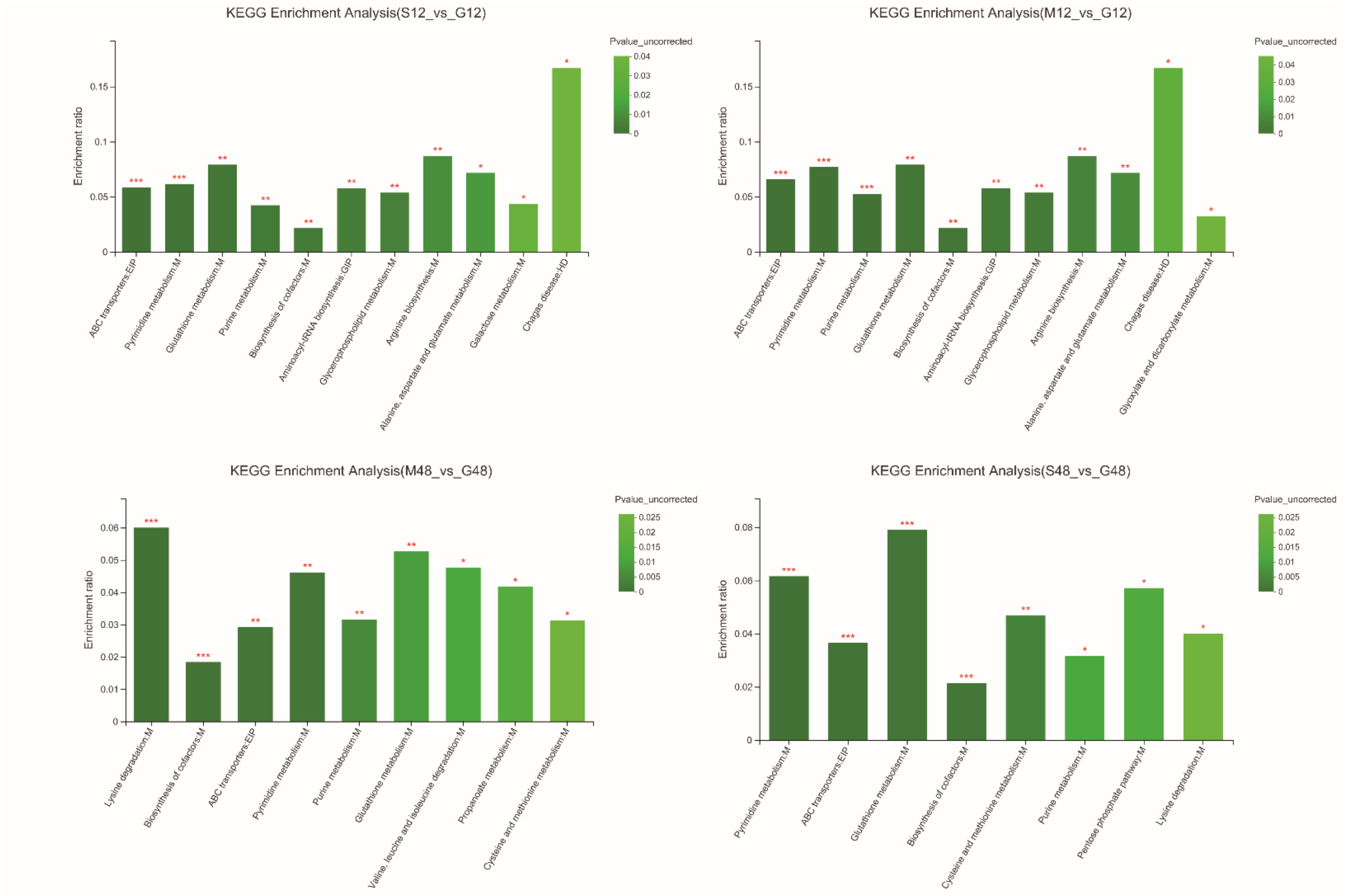

Figure S6: KEGG enrichment analysis of the different metabolites of different groups in *Crypthecodinium* sp. SUN. (A) ~ (D) were KEGG analysis of S12vsG12, M12vsG12, M48vsG48 and S48vsG48 respectively.
