# Supplementary material for: Mechanisms of Sodium-Acetate-Induced DHA Accumulation in a DHA-Producing Microalga, Crypthecodinium sp. SUN
Source: Mar Drugs. 2022 Aug 9;20(8):508. doi: 10.3390/md20080508 (PMC9409966; doi:10.3390/md20080508)
Supplement: Supplementary file 1 [file marinedrugs-20-00508-s001.zip › Supplementary file 2.pdf]

Table S1

Overview of DEG in different carbon sources in *Crypthecodinium* sp. SUN

group	total DEG	up	down
G0_vs_G6	4461	2185	2276
G0_vs_M6	3239	1443	1796
G0_vs_S6	2350	1010	1340
G0_vs_G12	3170	1687	1483
G0_vs_M12	3830	1743	2087
G0_vs_S12	2548	832	1716
G0_vs_G24	1729	1033	696
G0_vs_M24	2370	883	1487
G0_vs_S24	2404	853	1551
G0_vs_G48	1329	678	651
G0_vs_M48	2230	955	1275
G0_vs_S48	2992	900	2092

Table S2

Overview of all number of metabolites and different number of metabolites among three groups in *Crypthecodinium* sp. SUN

Name	G12_vs_G0	M12_vs_G0	S12_vs_G0	G48_vs_G0	M48_vs_G0	S48_vs_G0
all_number	514	514	514	514	514	514
diff_number	68	63	55	56	42	46
